# Investigating Protective Effect of Suspension of Paeoniflorin in Combination with Curcumin Against Acute Liver Injury Based on Inhibition of TLR4/NF-κB/NLRP3 Inflammatory Pathway

**DOI:** 10.3390/ijms26136324

**Published:** 2025-06-30

**Authors:** Zhengkun Wu, Yinquan Zhao, Yang Wang, Haohuan Li, Funeng Xu, Wei Zhang, Hualin Fu, Lizi Yin, Felix Kwame Amevor, Juchun Lin, Danqin Li, Gang Shu

**Affiliations:** 1 Department of Basic Veterinary Medicine, Sichuan Agricultural University, Chengdu 611130, China; fcm0417@163.com (Z.W.); paprikazyq@163.com (Y.Z.); 13350023912@163.com (Y.W.); lihaohuan7@163.com (H.L.); funengxu@sicau.edu.cn (F.X.); zhangwei26510c@126.com (W.Z.); fuhl.sicau@163.com (H.F.); yinlizi@hotmail.com (L.Y.); amevorfelix@gmail.com (F.K.A.); cnddwyx@163.com (J.L.); 2 College of Veterinary Medicine, Kansas State University, 1700 Denison Ave, Manhattan, KS 66502, USA

**Keywords:** paeoniflorin, curcumin, suspension, acute liver injury, TLR4/NF-κB/NLRP3

## Abstract

The objective of this study was to formulate a compound suspension comprising paeoniflorin and curcumin, assess its quality characteristics, and investigate its protective efficacy against acute liver injury in mice. The prescriptions were screened using a single-factor test, and nine groups of suspensions were prepared using the dispersion method. Fifty KM mice (four weeks old) were selected and randomly divided into five groups: the CON, LD, PF, CUR, and PC groups. The doses of both paeoniflorin and curcumin were 100 mg/kg BW, and different suspensions were given to different groups by gavage for 14 days. All the groups except the CON group were injected intraperitoneally with 20 μg/kg LPS and 700 mg/kg D-GalN on the last day. According to the results, the suspension prepared using the optimal prescriptions was orange-yellow in color, with homogeneous turbidity and good re-dispersibility. The combination treatment could reduce the severity of pathological injuries of liver, improve the ultrastructure of hepatocytes, increase the activities of T-SOD, GSH-Px, and CAT, decrease the levels of IFN-γ, TNF-α, and IL-1, and down-regulate the expression of genes such as TLR4, MyD88, IκBα, and NLRP3. The underlying mechanism might be associated with the enhancement of antioxidant enzyme activities, inhibition of the TLR4/NF-κB/NLRP3 signaling pathway, and suppression of inflammasome assembly and release in hepatic tissues.

## 1. Introduction

Paeoniflorin is one of the main bioactive components extracted from the roots of *Paeonia lactiflora* and *Paeonia × suffruticosa*. Its medicinal source, Radix Paeoniae Alba (white peony root), has been used for millennia in the treatment of liver pain and immune disorders. Numerous studies have shown that paeoniflorin can exert various pharmacological effects such as hepatoprotection, cardiovascular protection, neuroprotection, immunomodulation, anti-inflammation effects, and paroxysmal pain relief by regulating immune cells and acting on the PI3K/Akt/mTOR pathway and MAPKs/NF-κB pathway [1,2,3]. Curcumin is a polyphenolic compound extracted and isolated from *Curcuma longa*, which has pharmacological activities including antioxidant, lipid regulation, antibacterial, antiviral, and anticancer effects. With the further development of modern research, curcumin has been applied to the treatment of oxidative liver injury due to its low toxicity, good antioxidant activity, and free radical scavenging ability. It also has wide-ranging potential applications in the food industry, pharmaceuticals, and nutraceuticals [4,5,6].

However, although both paeoniflorin and curcumin can exert significant hepatoprotective effects through their anti-inflammatory and antioxidant pharmacological activities [7,8], there have been no studies reporting on their combined application. Considering the different solubility of paeoniflorin and curcumin, we decided to prepare them as a complex suspension in order to facilitate the simultaneous application of these two drugs. Suspensions can improve the oral bioavailability and stability of hydrophobic drugs; by dispersing curcumin particles in an aqueous carrier dissolved with paeoniflorin and then stabilized using surfactants and flocculants, we could achieve simultaneous and precise drug delivery [9]. A lipopolysaccharide (LPS)/D-galactosamine (D-GalN)-induced acute liver injury model in mice has been widely used to assess the efficacy of hepatoprotective drugs. The LPS/D-GalN-induced model is associated with more intense inflammation, more severe cellular metabolism disruption, and a more concentrated injury in the liver compared to an LPS-induced model due to the fact that D-GalN, as a specific hepatic sensitizer, inhibits the transcriptional activity of cells [10]. Up to now, this model of acute liver injury has been used to study the hepatoprotective mechanisms of several natural drug monomers and their derivatives, including isoglycyrrhizin, silymarin, and quercetin; the hepatoprotective effect of paeoniflorin in combination with curcumin was explored in this study with the help of this model [11,12,13].

A test of the preparation used in this study showed that the compound suspension was turbid and uniform and did not settle easily, with good re-dispersibility. Animal experiments demonstrated that paeoniflorin in combination with curcumin could effectively protect the liver; the mechanism may lie in the enhancement of hepatic antioxidant enzyme activities, the inhibition of TLR4/NF-κB signaling pathway activation, and the blockage of inflammasome assembly and release. The above results provide a formulation basis for the combination of paeoniflorin and curcumin, clarify their hepatoprotective efficacy and mechanism of action in an LPS/D-GalN-induced acute liver injury model, expand the understanding of this suspension’s development and application, and lay the foundation for subsequent research.

## 2. Results

### 2.1. Appearance and Quality of Different Groups of Compound Suspensions

The experimentally prepared mixtures containing paeoniflorin and curcumin were orange-yellow in color, with an overall uniform turbidity and no Tyndall effect. The upper layer of the liquid was clear and transparent after static delamination, and the lower layer of the precipitate was orange-yellow in color. The sedimentation volume ratio (F) within 7 days after preparation and the number of re-dispersions (N) after 7 days are shown in Table 1.

### 2.2. Protective Effect of Different Suspensions on Body Weight and Organ Index

**Figure 1:**
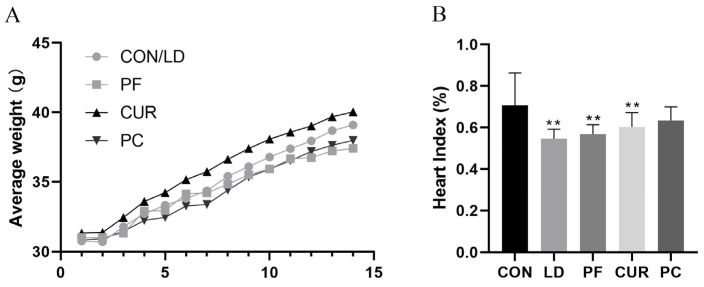

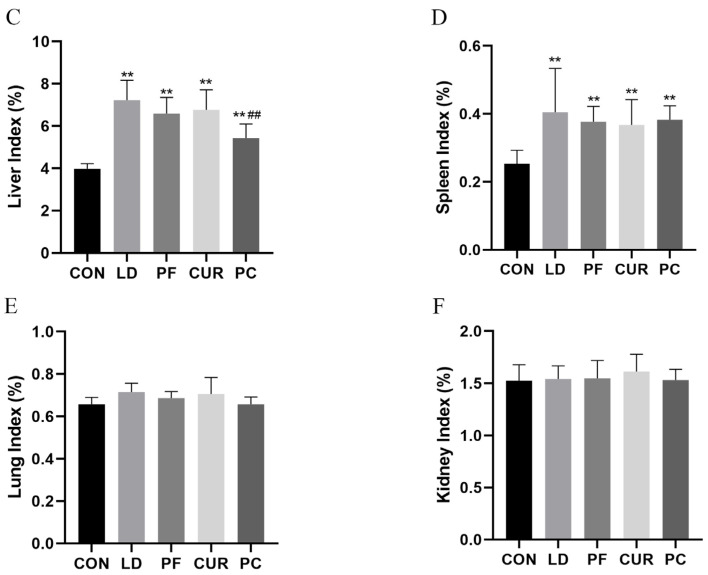
Weights and organ indexes. (**A**) Average weight; (**B**) heart index; (**C**) liver index; (**D**) spleen index; (**E**) lung index; (**F**) kidney index. Data are presented as means ± SD. ** *p* < 0.01 vs. CON group; ## *p* < 0.01 vs. LD group. CON: control group; LD: LPS/D-GalN (model) group; PF: paeoniflorin; CUR: curcumin; PC: paeoniflorin combined with curcumin.

The changes in average weight and organ indexes are shown in Figure 1. During the test cycle, the body weight of all the mice showed an upward trend, in which the mice in the CON and LD groups showed stable growth in their body weight without obvious fluctuations. The mice in the PF group showed a similar growth rate to that of the CON group in the first 7 days, and then their growth rate slowed down in the second 7 days, and they had the lowest body weight at the end of the test. The mice in the CUR group showed a higher body weight growth rate and had the highest body weight at the end of the test. The mice in the PC group showed a lower body rate growth rate in the first 7 days, and then their growth rate increased in the last 7 days. Their growth rate was elevated, and their body weight at the end of the experiment was higher than that of the PF group but lower than that of the CON group. The organ indexes calculated using the corresponding formula showed that compared with the CON group, the heart index of the mice in the LD, PF, and CUR groups was highly significantly lower (*p* < 0.01), and the liver and spleen indexes of the mice in the LD, PF, CUR, and PC groups were highly significantly higher (*p* < 0.01). Compared with the LD group, the mice in the PC group showed a highly significant (*p* < 0.01) decrease in their liver index, while no significant changes were observed in the other organ indexes, including those for the lungs and kidneys.

### 2.3. The Protective Effect of Different Suspensions on the Liver’s Histopathologic Structure

**Figure 2:**
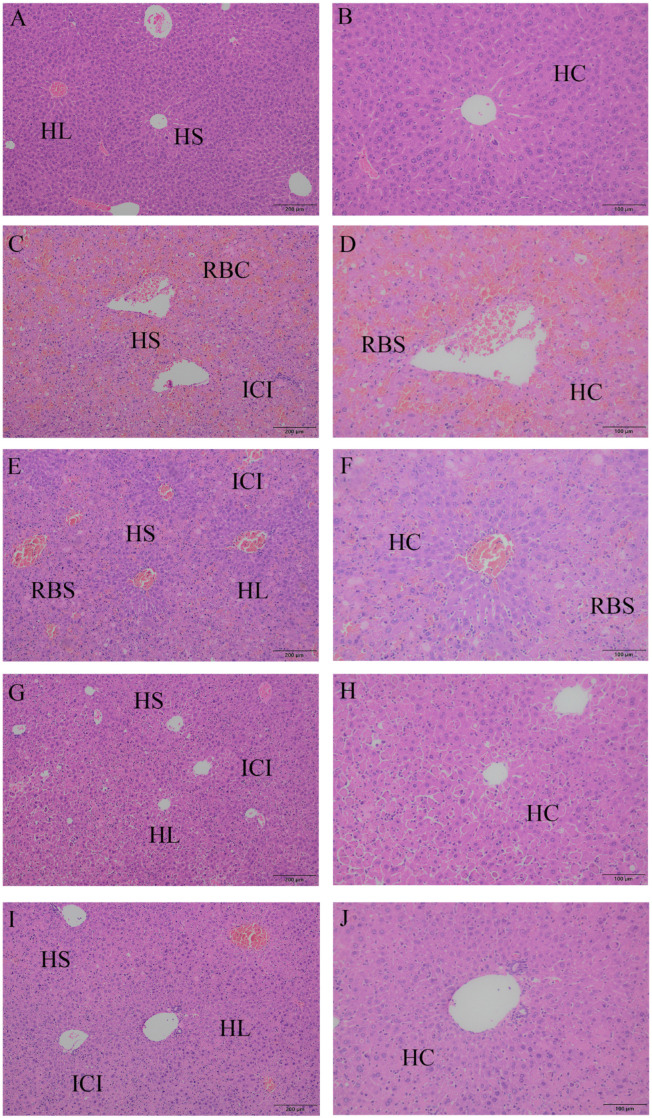
Liver morphology histopathological observation. Left magnification: 200 μm; right magnification: 100 μm. (**A**,**B**) CON group liver; (**C**,**D**) LD group liver; (**E**,**F**) PF group liver; (**G**,**H**) CUR group liver; (**I**,**J**) PC group liver. HL: hepatic lobule; HS: hepatic sinusoid; HC: hepatic cell; ICI: inflammatory cell infiltration; RBC: red blood cell.

Through HE staining and light microscopy (Figure 2), we could observe that the hepatocytes in the CON group were arranged radially, the structure of the liver lobules was normal, there was no visible inflammatory cell infiltration or vascular dilatation, and the hepatic blood sinusoids had clear shapes, with no obvious dilatation or occlusion. The morphology of the hepatocytes was regular, the cytoplasm was eosinophilic and homogeneously stained, the nucleus was located in the center of the cell, the staining was uniform, and no obvious vacuoles or necrosis was seen. In the LD group, the arrangement of the hepatocytes was disordered, the structure of the hepatic lobules was ambiguous, there was a large amount of neutrophil and monocyte infiltration as well as vascular dilatation, the hepatic blood sinusoids were obviously dilated, and there was a large amount of erythrocyte filling. The hepatocytes showed obvious swelling and balloon-like changes and cytoplasmic laxity, and some cells showed nuclear consolidation, nuclear fragmentation, and nuclear lysis, suggesting cell necrosis. The hepatocytes in the PF group and the CUR group were arranged in a more regular manner, with hepatic lobules with a relatively normal structure, and there was inflammatory cell infiltration, vasodilatation, the dilatation of the hepatic sinusoids, and erythrocyte filling, but these were less severe than those in the LD group. The hepatocyte contours were relatively complete, ballooning and necrosis were significantly reduced, the cytoplasmic staining was more uniform than that of the LD group, and a small number of cells showed nuclear consolidation. The hepatocytes in the PC group were regularly arranged, the hepatic lobular structure was clear, a very small amount of inflammatory cell infiltration was seen around the central vein, the blood vessels and hepatic blood sinusoids were slightly dilated, and no obvious erythrocyte filling was seen. The morphology and staining of the hepatocytes were not different from those of the CON group, and only a very few cells had mild nuclear consolidation.

### 2.4. The Protective Effect of Different Suspensions on the Liver’s Ultrastructure

**Figure 3:**
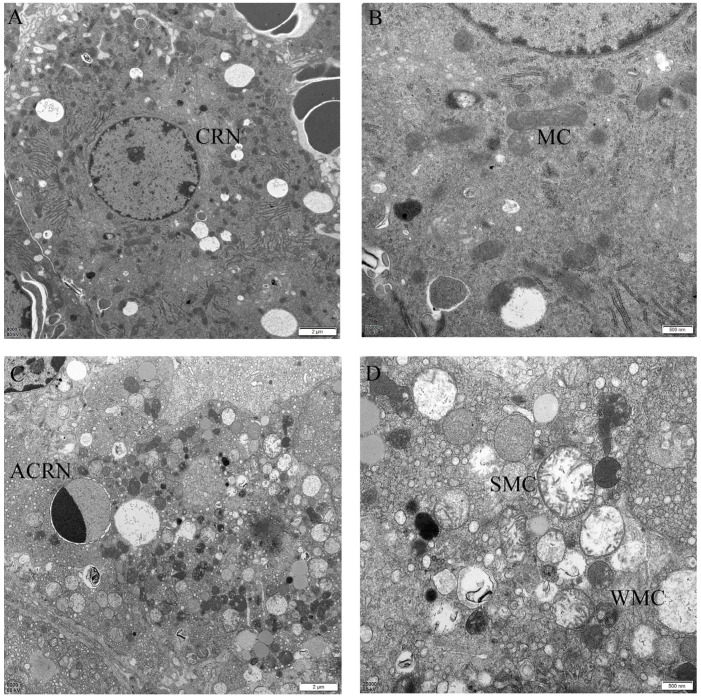

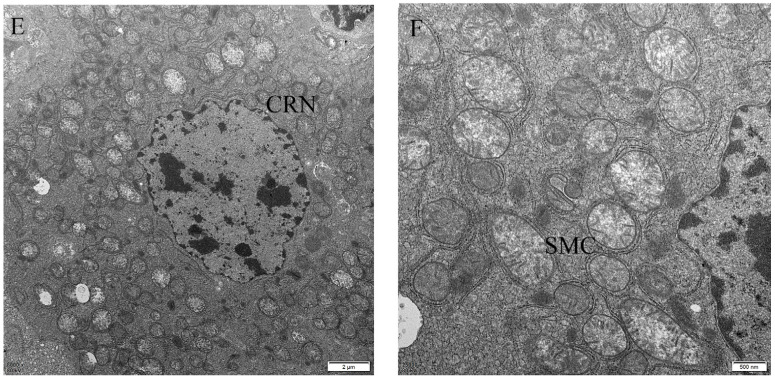
Liver ultrastructure observation. Left magnification: 2 μm; right magnification: 500 nm. (**A**,**B**) CON group liver; (**C**,**D**) LD group liver; (**E**,**F**) PC group liver. CRN: cell nuclei; ACRN: apoptotic cell nuclei; MC: mitochondria; SMC: swollen mitochondria; WMC: wrinkled mitochondria.

The ultrastructure of the liver was observed by transmission electron microscopy (TEM) showed in Figure 3. Under a low magnification, we observed that the cell structure of the CON group was normal, the nuclear membrane was rounded and regular with no deformation and the boundary with the cytoplasm was clear, the chromatin in the nucleus was loose and irregularly distributed, and the number of lipid droplets was normal, with the droplets randomly dispersed in the cytoplasm. The cell structure of the LD group was disordered, and despite the regularity of the nuclear membrane, the boundary with the cytoplasm was unclear, and the chromatin in the nucleus was condensed on one side, which suggested the apoptotic death of the cells, and the number of lipid droplets was obviously reduced. In the PC group, the cells were more normal, the nuclear membrane was irregular but clearly demarcated from the cytoplasm, the chromatin in the nucleus was similar to that in the CON group, and the number of lipid droplets was reduced but higher than that in the LD group. Under a high-magnification microscope, we observed that the mitochondria of the CON group were normal in structure and irregularly oval, with clear mitochondrial cristae inside. The mitochondria of the LD group were obviously swollen and spherical, and some of the mitochondria were wrinkled with fewer cristae. The mitochondria of the PC group had different degrees of swelling and wrinkling but less than the LD group.

### 2.5. Protective Effect of Different Suspensions on Biochemical Indices

**Figure 4:**
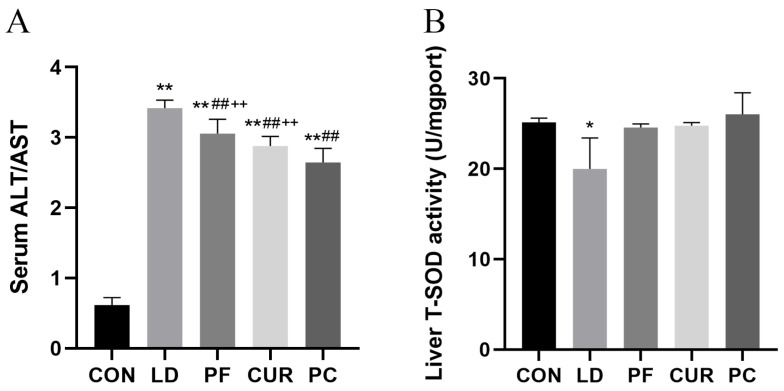

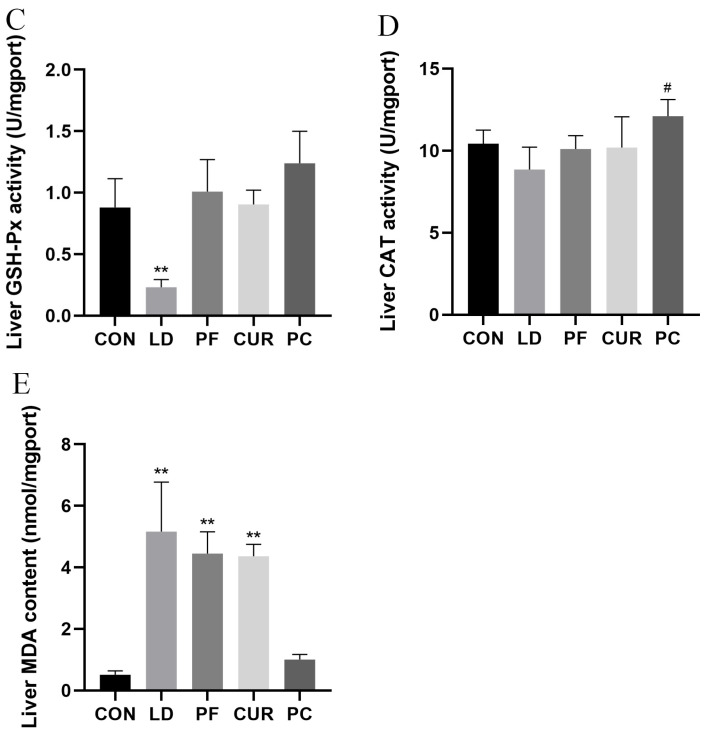
Biochemical indicator testing. (**A**) Serum ALT/AST; (**B**) liver T-SOD; (**C**) liver GSH-Px; (**D**) liver CAT; (**E**) liver MDA. * *p* < 0.05 vs. CON group; ** *p* < 0.01 vs. CON group; # *p* < 0.05 vs. LD group; ## *p* < 0.01 vs. LD group; ++ *p* < 0.01 vs. PC group.

The results of the serum and liver biochemical tests are shown in Figure 4. Compared with the serum of the CON group, the ALT/AST in the serum of the LD group, PF group, CUR group, and PC group were extremely significantly higher (*p* < 0.01). The ALT/AST was highly significantly lower (*p* < 0.01) in the serum of the PF, CUR, and PC groups compared to that of the LD group. Compared with the PC group, the ALT/AST was extremely significantly higher in the serum of the PF and CUR groups (*p* < 0.01). Compared with the livers of the CON group, the MDA content in the livers of the LD, PF, and CUR groups was extremely significantly higher (*p* < 0.01), the T-SOD activity in the livers of the LD group was significantly lower (*p* < 0.05), and the GSH-Px activity was extremely significantly lower (*p* < 0.01) in the livers of the CUR and PC groups. Compared with the livers of the LD group, the livers of the PC group showed significantly higher CAT activity (*p* < 0.05).

### 2.6. Protective Effect of Different Suspensions on Serum Factors

**Figure 5:**
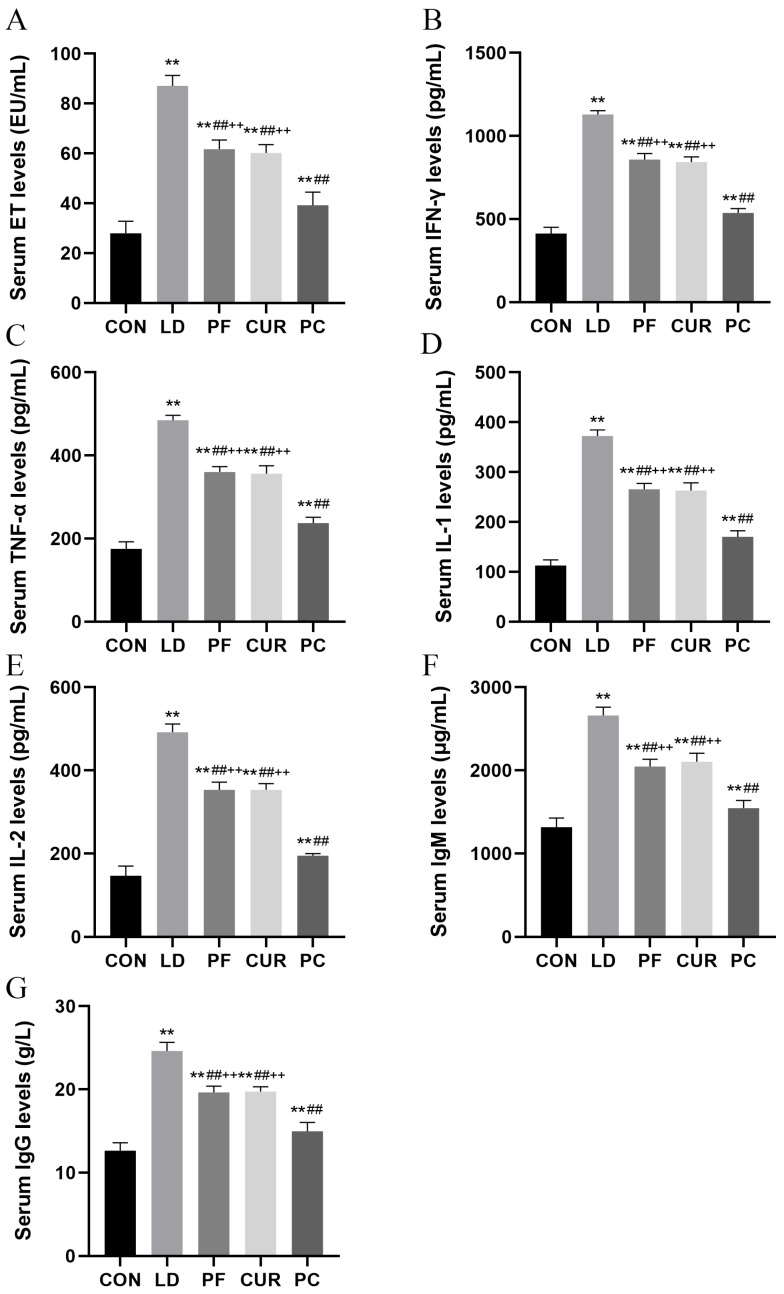
Serum ELISA testing. (**A**) ET; (**B**) IFN-γ; (**C**) TNF-α; (**D**) IL-1; (**E**) IL-2; (**F**) IgM; (**G**) IgG. ** *p* < 0.01 vs. CON group; ## *p* < 0.01 vs. LD group; ++ *p* < 0.01 vs. PC group.

The serum ELISA results are shown in Figure 5. Compared with the serum of the CON group, the serum of the LD group, PF group, CUR group, and PC group contained highly significantly higher levels of ET, IFN-γ, TNF-α, IL-1, IL-2, IgM, and IgG (*p* < 0.01). The serum levels of ET, IFN-γ, TNF-α, IL-1, IL-2, IgM, and IgG were highly significantly lower in the PF, CUR, and PC groups compared with the LD group (*p* < 0.01). The serum levels of ET, IFN-γ, TNF-α, IL-1, IL-2, IgM, and IgG were highly significantly higher in the PF group compared to the PC group (*p* < 0.01).

### 2.7. Protective Effects of Different Suspensions on Liver mRNA Expression

**Figure 6:**
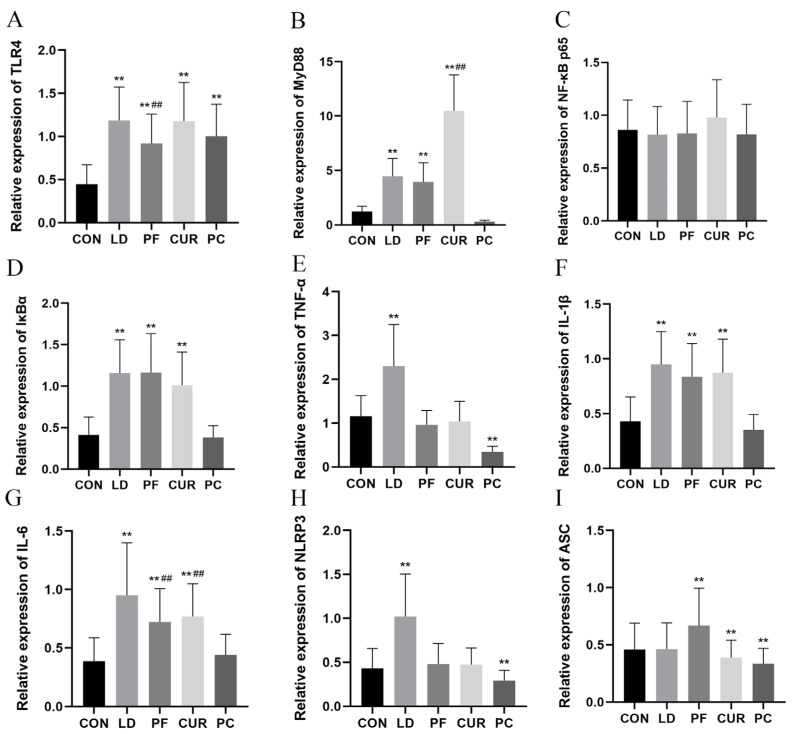
Liver—relative gene expression. (**A**) TLR4; (**B**) MyD88; (**C**) NF-κB; (**D**) IκBα; (**E**) TNF-α; (**F**) IL-1β; (**G**) IL-6; (**H**) NLRP3; (**I**) ASC. ** *p* < 0.01 vs. CON group; ## *p* < 0.01 vs. LD group.

The results of a real-time fluorescence quantitative (RT-qPCR) assay are shown in Figure 6. Compared with the livers of the CON group, the expression of TLR4 in the livers of the LD group, the PF group, the CUR group, and the PC group was extremely significantly higher (*p* < 0.01); the expression of MyD88, IκBα, IL-1β, and IL-6 in the livers of the LD group, the PF group, and the CUR group was extremely significantly higher (*p* < 0.01); the expression of ASC was highly significantly lower in the livers of the CUR group and the PC group (*p* < 0.01); the expression of TNF-α and NLRP3 was highly significantly higher in the livers of the LD group (*p* < 0.01); the expression of ASC was highly significantly higher in the livers of the PF group (*p* < 0.01); and the expression of TNF-α and NLRP3 was highly significantly lower in the livers of the PC group (*p* < 0.01). Compared with the LD group, the expression of IL-6 in the livers of the PF group and the CUR group was extremely significantly lower (*p* < 0.01); the expression of TLR4 in the livers of the PF group was extremely significantly lower (*p* < 0.01); and the expression of MyD88 in the livers of the CUR group was extremely significantly higher (*p* < 0.01). However, no significant changes were observed in the expression of NF-κB p65 in the livers of all the groups.

## 3. Discussion

In this research, we screened and optimized the prescription of a compound suspension containing paeoniflorin and curcumin. Based on the optimal prescription, we prepared the suspension, performed quality control, and conducted animal experiments to elucidate the protective effect of the compound suspension on an LPS/D-GalN-induced model by exploring the changes in the body weight, organ indexes, liver histology, hepatocyte ultrastructure, antioxidant enzyme activities, inflammatory cytokine levels, immunoglobulin levels, and the TLR4/NF-κB/NLRP3 pathway.

In the present study, the traditional Chinese medicine formulas of White Peony Root and Turmeric Soup and a Liver Pain Relief Pill were referenced to combine paeoniflorin and curcumin, the main components of white peony root and turmeric. Paeoniflorin is commonly dissolved in saline and gavaged [14], and curcumin is often gavaged with 0.5% sodium carboxymethylcellulose (CMC-Na) as a carrier [15]. Typically, an experiment using two large doses of a drug implies the need to prepare a high, unstable concentration of the drug and the need to gavage the mice twice a day. In order to ensure the accuracy of the test and to safeguard the welfare of the experimental animals, paeoniflorin and curcumin were made into a compounded suspension with reference to the Compound Sulphur Lotion. The results of an orthogonal test showed that the suspensions in groups 7, 8 and 9 were stable and did not easily settle within 7 days; the sedimentation volume ratio (F) was consistent with the results for other similar prescriptions [16], in accordance with the quality standard for suspensions stipulated in the Chinese Veterinary Pharmacopoeia (CVP), and the compound had good re-dispersibility, and group 9 was chosen to be the final prescription for the compounded suspensions.

Existing reports claim that paeoniflorin not only exerts sedative–hypnotic effects [17] but also prevents high-fat-diet-induced nonalcoholic fatty liver disease (NAFLD) by regulating lipid metabolism [18]. Curcumin enhances the growth performance of animals and promotes weight gain, due to which it has been widely used in aquaculture [19]. In this experiment, the body weight of all the mice showed an increasing trend; the CON group showed a steady weight increase, the PF group showed the slowest weight increase, and the CUR group showed the fastest weight increase, which was in line with the above findings. The final body weight of the PC group was higher than that of the PF group but lower than that of the CON group, and it was hypothesized that paeoniflorin had a higher regulating effect on the body weight than curcumin at the same dosage. The organ index is the ratio of the absolute weight of organs to the body weight, which can reflect the size, function, and health status changes of organs, and an acute liver injury induced by LPS combined with D-GalN can cause an elevated organ index for the liver and spleen [20]. In this experiment, all groups receiving an LPS/D-GalN-induced injury had highly significant elevated liver and spleen organ indexes, which was consistent with what was reported in the literature, and the heart organ indexes were highly significantly lower in the LD, PF, and CUR groups, which may have been related to dehydration due to acute inflammation [21]. In addition, the liver organ index was smaller in the PC group than in the PF and CUR groups, and the heart organ index was not different from that of the CON group, and the above results suggest that a combination of paeoniflorin and curcumin may exert a better organ-protective effect at this dose.

As a branch of pathology, histopathology can observe changes in the tissue structure and cell morphology in disease states using light microscopy, which can reflect the type, nature, and course of many diseases. The results of this study indicated that the livers of the CON group showed normal features, from the hepatic lobules to the hepatocytes. The livers of the LD group showed the obvious destruction of the structure of the hepatic lobules, the infiltration of neutrophils and monocytes, the dilation of the hepatic sinusoids, a large amount of erythrocyte filling, the ballooning of hepatocytes, nuclear consolidation, and other phenomena that conformed to the histopathological features of an LPS-induced acute liver injury model that have been reported [22,23]. Both the PF and CUR groups showed an improvement compared with the LD group but were still some distance away from the normal level of the CON group, which indicated that both paeoniflorin and curcumin at this dose could protect against LPS-induced acute liver injury but were not enough to completely counteract its effects, a result consistent with literature reports [7,8]. The PC group administered combined paeoniflorin and curcumin almost recovered to the level of the CON group and exerted a stronger protective effect than either drug alone, suggesting that the combination of the two has more potential.

TEM is a microscopic technique that utilizes an electron beam to penetrate an ultrathin sample and form a highly magnified image on a fluorescent screen or an imaging system and is capable of showing the intracellular ultrastructure. According to existing reports [24], the nuclear membrane of normal hepatocytes observed using electron microscopy is rounded and clearly demarcated, the chromatin in the nucleus is loose, and the mitochondria are irregularly elliptical, and the electron microscopy results for the CON group in this experiment were consistent with them. The nuclear membrane boundary of the LD group was blurred, and the chromatin in the nucleus was condensed along the periphery of the nuclear membrane to form apoptotic vesicles, which is a phenomenon in line with the findings of related studies [25]. In addition, a large number of swollen and morphologically disorganized mitochondria were present, suggesting severe mitochondrial damage, which is a typical feature of acute liver injury and is consistent with the results of an existing study that also used an LPS/D-GalN-induced model [26]. The literature suggests [27] that a decrease in the mitochondrial membrane potential during the early apoptotic phase drives mitochondrial matrix condensation, which provides a basis for the mitochondrial crumpling observed in the LD group. The PC group showed a largely normal cellular structure, the absence of significant apoptosis, and less mitochondrial swelling and crumpling. This implies that the combination of the two drugs at this dose has a certain hepatoprotective effect; this effect may be associated with the anti-inflammatory and antioxidant potency of both drugs.

The serum alanine aminotransferase (ALT) and aspartate aminotransferase (AST) levels are important indicators for assessing liver function, and when acute liver injury occurs, the ALT and AST levels will rise rapidly and the ALT will often be higher than the AST [28]. The results showed that the serum ALT/AST in the LD group was much higher than that in the CON group, indicating that the liver was severely injured in the LD group and the model of acute liver injury was successfully established. The serum ALT/AST in the PF group and the CUR group was lower than that in the LD group, which indicated that both paeoniflorin and curcumin at the same dosage could ameliorate the LPS-induced acute liver injury; both of them were hypothesized to have a certain protective effect on the integrity of the liver cells, and all of the results were consistent with the existing studies [7,8,26]. The serum ALT/AST in the PC group was extremely significantly lower than that in the PF and CUR groups, suggesting that the combination of the two at this dose had a better protective effect on hepatocytes. Total superoxide dismutase (T-SOD), glutathione peroxidase (GSH-Px), and catalase (CAT) are antioxidant enzymes that respond to the ability of tissues to scavenge various types of reactive oxygen species (ROS). Malondialdehyde (MDA) is a lipid peroxidation end-product, the content of which is closely related to oxidative damage to cells [29]. In this study, we found that the T-SOD, GSH-Px, and CAT activities in the livers of the LD group showed a decreasing trend and the MDA content was highly significantly increased compared with that in the CON group, which is consistent with the results of the existing reports, indicating that LPS-activated inflammation induced ROS generation, which led to increased oxidative stress in the liver [30,31]. On the other hand, the T-SOD, GSH-Px, and CAT activities in the liver of the PF and CUR groups were elevated compared with those in the LD group and almost indistinguishable from those of the CON group, and the MDA content also showed a decreasing trend, which is consistent with the relevant reports on the hepatoprotective effects of paeoniflorin and curcumin, suggesting that both of them can exert a certain antioxidant effect and assist in the scavenging of ROS [7,8]. In addition, the T-SOD, GSH-Px, and CAT activities and MDA content in the livers of the PC group were restored to the levels of the CON group, suggesting that the combination of the two drugs at this dose exerted an extremely significant antioxidant effect and greatly alleviated the oxidative stress in the liver.

Endotoxin (ET) refers to an LPS that can cause a strong inflammatory response, and when it invades the body, innate immune cells such as macrophages and dendritic cells recognize pathogen-associated molecular models (PAMPs) using pattern recognition receptors (PRRs) such as Toll-like receptors (TLRs) and NOD-like receptors (NLRs), activate signaling pathways such as the NF-κB and MAPK pathways, and produce a series of inflammatory mediators such as interferon-γ (IFN-γ), tumor necrosis factor α (TNF-α), and interleukin 1 (IL-1) [32,33]. At the same time, dendritic cells activated by PAMPs present pathogenic fragments to T cells, which initiate the proliferation and differentiation of B cells through a series of immune responses and release a large number of specific antibodies [34]. Among these antibodies, immunoglobulin M (IgM), as a product of the primary immune response, can rapidly polymerize antigens and activate the complement system to limit the spread of pathogens, and immunoglobulin G (IgG), secreted by memory B cells in large quantities during the secondary immune response, can provide highly effective and long-lasting protection [35]. In this experiment, the serum levels of ET, IFN-γ, TNF-α, IL-1, interleukin 2 (IL-2), IgM, and IgG in the LD group were all extremely significantly increased compared with those of the CON group, and this result was attributed to the inflammatory and immune responses generated by the LPS/D-GalN-induced acute liver injury. Although a correlation between the levels of LPS and IL-2 has not been reported, considering that IL-2 acts as an inflammatory cytokine that can play a role in the early immune response, this result is reasonable. In addition, compared with the LD group, the results of all the assays in the PF and CUR groups were significantly decreased, and it was hypothesized that both paeoniflorin and curcumin at this dose could act on the inflammation-related signaling pathway and reduce the production of inflammatory mediators, which is in line with the results of existing studies [7,8]. The serum indicators in the PC group decreased extremely significantly compared with those in the PF and CUR groups and recovered to a level slightly higher than that of the CON group, indicating that the combination of these two drugs can better reduce the secretion of inflammatory cytokines and play a greater role in the clearance of ET, resulting in relatively milder immune responses and the production of relatively fewer specific antibodies. Considering that LPS can generate ROS and exacerbate oxidative stress by activating NADPH enzymes and damaging mitochondria and ROS can induce the release of inflammatory cytokines by activating the NF-κB signaling pathway and facilitating the assembly of NLRP3 inflammatory vesicles [36], it is hypothesized that the combination of these two drugs may exert anti-inflammatory effects by protecting mitochondria, scavenging reactive oxygen species, and acting directly on inflammation-related pathways.

Existing studies have shown that TLR4 recruits MyD88 to the cytoplasmic domain upon the recognition of PAMPs such as LPS, and MyD88 interacts with interleukin 1 receptor-associated kinase 4 (IRAK4), which prompts the phosphorylation and degradation of inhibitory factor κBα (IκBα). Then, IκBα binds to NF-κB p65, the core subunit of NF-κB, and causes the translocation of the released NF-κB into the nucleus, initiating the transcription of inflammatory genes such as TNF -α, IL-1β, and IL-6 and others [37,38]. Not only that but NF-κB also up-regulates the gene expression of NLRP3, pro-IL-1β, and pro-IL-18 during the initiation phase of inflammatory vesicle production, providing protein substrates and transcription products for the activation phase of inflammatory vesicle production. ROS accumulation, a K^+^ efflux, and other stimuli can trigger NLRP3’s oligomerization or polymerization with the adaptor protein ASC through PYD interaction and the recruitment and activation of effector enzyme caspase-1 to form inflammatory vesicles, which leads to the maturation and secretion of IL-1β. What is more, IL-1β in turn can reactivate NF-κB through its receptor (IL-1R) to form a positive feedback loop and amplify the inflammatory response, together with inflammatory mediators such as TNF-α [39,40]. In this experiment, there was no significant difference in the expression of NF-κB p65 in the livers of all the groups. This was attributed to the fact that the key regulation of the NF-κB signaling pathway occurs at the protein level and does not depend on mRNA changes in the p65 subunit and that the expression of NF-κB p65 is relatively stable in most tissues and is usually unchanged [41]. Although it was mentioned above that the protein level of IκBα decreases due to phosphorylation hydrolysis when inflammation occurs, it has been reported that NF-κB itself up-regulates the transcription of IκBα, which compensates for the loss of the protein through negative feedback and inhibits the NF-κB signaling pathway and inflammatory response. This effect ultimately manifests in the up-regulation of the mRNA level of this gene [42], which provided highly significant up-regulation of the expression of IκBα in the livers of the LD group. Meanwhile, the expression levels of TLR4, MyD88, TNF-α, IL-1β, IL-6, and NLRP3 in the livers of the LD group were all extremely significantly increased compared with those in the CON group, which implies that LPS exacerbates inflammation through the MyD88-dependent TLR4 pathway, the ROS stimulation of inflammatory vesicles, and other mechanisms. Comparing the PF group with the LD group, this experiment observed a highly significant decrease in the gene expression of TLR4 in the livers of the PF group, suggesting that paeoniflorin can down-regulate TLR4, a finding consistent with existing reports [43,44]. The experiment also observed a down-regulation of the expression of NLRP3 and an up-regulation of the expression of ASC in the livers of the PF group, consistent with relevant reports on the limited antioxidant effect of paeoniflorin [45]; this was suspected to cause the ROS-dependent stimulation of inflammatory vesicles with weak effects. Considering the highly significant reduction in the expression levels of TNF-α and IL-6, it is hypothesized that paeoniflorin exerts its anti-inflammatory effects mainly by affecting the expression of TLR4 and inhibiting the downstream NF-κB pathway and that the anti-inflammatory effects are much greater than the antioxidant effects. In contrast, in the livers of the CUR group the IL-6 expression was significantly down-regulated, the NLRP3 and TNF-α expression was restored to the level of the CON group, and the ASC expression was lower than the level of the CON group, which suggests that curcumin may play an anti-inflammatory role by affecting the assembly of inflammatory vesicles. However, there was no significant change in the expression of TLR4, and the expression of MyD88 was extremely up-regulated, which was inconsistent with the existing reports [46]. This may have been related to the fact that curcumin inhibited downstream ROS production, NLRP3 conformational changes were blocked, and cells tended to up-regulate the transcription of TLR4 and MyD88 to enhance the stimulation of inflammatory vesicles and restore their assembly. Based on the results of the above experiments, it is hypothesized that curcumin exerts its anti-inflammatory effects by scavenging ROS through its antioxidant activity and inhibiting the activation of inflammatory vesicles [47]. In addition, this study found that in the PC group the hepatic TLR4 expression was down-regulated, the MyD88, IκBα, IL-1β, and IL-6 expression was restored to the level of the CON group, and the TNF-α expression was even lower than normal, which implies that paeoniflorin coupled with curcumin can more significantly affect the TLR4/MyD88 pathway and reduce the degradation of IκBα, the release and translocation of NF-κB, and the downstream expression of inflammatory genes, inhibiting the NF-κB signaling pathway more strongly and exerting a better anti-inflammatory effect than one drug alone. Not only that but the hepatic NLRP3 and ASC expression in the PC group was lower than the level in the CON group, indicating that the combination of the two played an inhibitory role in both the initiation and activation of inflammatory vesicles. It was hypothesized that this anti-inflammatory effect was caused by decreasing the gene regulation of the initiation phase by NF-κB and scavenging ROS to reduce their stimulation of the activation phase of NLRP3.

## 4. Materials and Methods

### 4.1. Chemicals

Paeoniflorin, curcumin, CMC-Na, Tween 80, citric acid, sodium benzoate, LPS, D-GalN, total superoxide dismutase (T-SOD, A001-1-2), catalase (CAT, A007-1-1), glutathione peroxidase (GSH-Px, A005-1-2), malondialdehyde (MDA, A003-1-2), and total protein (TP, A045-2-2) kits were purchased from the Jiancheng Bioengineering Institute, Nanjing, China. Endotoxin (ET), interferon-γ (IFN-γ), tumor necrosis factor α (TNF-α), interleukin 1 (IL-1), interleukin 2 (IL-2), immunoglobulin M (IgM), and immunoglobulin G (IgG) ELISA kits were purchased from the Duma Biotechnology Corporation, Shanghai, China.

### 4.2. Preparation Method and Suspension Prescription Screening

Based on the results of the single-factor test, it was determined that the contents of paeoniflorin and curcumin in the prescription were 0.250 g. CMC-Na was selected as a suspending agent, Tween 80 as a wetting agent, citric acid as a flocculating agent, and sodium benzoate as an antioxidant. An L9 (3^4^) orthogonal method was chosen to design an orthogonal test to screen the dosages of CMC-Na, Tween 80, citric acid, and sodium benzoate. The factors and levels in the orthogonal test of the suspension prescription are shown in Table 2. The steps were as follows: Accurately weigh or measure paeoniflorin, curcumin, and the required excipients listed above, dissolve or swell paeoniflorin and the various excipients in distilled water and prepare them for use. Using the dispersion method, grind the curcumin thoroughly in a mortar, add the Tween 80 solution, and continue grinding until a fine paste is formed. Next, add the CMC-Na solution and grind it into the paste, transfer the mixture into a stoppered measuring cylinder, and rinse the mortar several times with distilled water. Then, transfer the rinsing solution, paeoniflorin solution, citric acid solution, and sodium benzoate solution into the same cylinder. Finally, adjust the total volume to 25 mL with distilled water, stopper the cylinder, and invert it several times to obtain a compound suspension of paeoniflorin and curcumin [48].

### 4.3. Quality Evaluation of Suspensions

Twenty-five milliliters of each of the 9 groups of suspensions, prepared using the orthogonal factors and levels given in Section 4.2, was put into stoppered measuring cylinders. After tightly sealing the cylinders, they were shaken vigorously for 1 min; the original volume of each group was recorded as V_0_. After standing at room temperature for 1 h, 2 h, 3 h, 6 h, 1 day, 2 days, 3 days, 4 days, 5 days, 6 days, and 7 days, the sediment volume of each group was recorded as V. The sedimentation volume ratio (F) was calculated using the following formula:

F = V/V_0_

In addition, the appearance, color, clarity, and number of re-dispersions (N) after 7 days were observed and recorded for each group of suspensions.

### 4.4. Animals and Experimental Design

Fifty healthy (4-week-old) male KM mice were purchased from Dashuo Biotechnology Co., Ltd., Chengdu, China—Animal License No.: SCXK (chuang) 2020-030—and the use of animals was approved by the Committee for the Protection and Utilisation of Animals of Sichuan Agricultural University (Approval No. 20240687). The mice were kept throughout the whole period in a standard SPF facility, with the ambient temperature controlled at 25 ± 2 °C and the ambient humidity controlled at 60 ± 5%, and fed ad libitum on clean drinking water and pellet feed (Dashuo Biotechnology Co., Ltd., Chengdu, China) under a 12/12 h light/dark cycle. After 5 days of acclimatization, the animals were randomly divided into five groups (*n* = 10): the control group (CON group), LPS/D-GalN (model) group (LD group), paeoniflorin group (PF group), curcumin group (CUR group), and paeoniflorin combined with curcumin group (PC group). The doses of both paeoniflorin and curcumin were 100 mg/kg BW [49,50,51,52]; the CON and LD groups were gavaged with a blank suspension, the PF group was gavaged with a suspension containing only paeoniflorin, the CUR group was gavaged with a suspension containing only curcumin, and the PC group was gavaged with the compounded suspension, with the maximal gavage amount being 0.2 mL/10 g/d. The test lasted for 14 days, and the changes in the body weight of the mice in each group were followed. At the end of the test, all the groups except the CON group were injected intraperitoneally with 20 μg/kg LPS and 700 mg/kg D-GalN [53,54], and the CON group was injected intraperitoneally with an equal amount of saline. All the mice were weighed and injected intraperitoneally with 80 mg/kg of 2% sodium pentobarbital 6 h later. Blood was collected from the orbital veins of the mice while they were anesthetized [s1].

### 4.5. Sample Collection

Blood was collected in 1.5 mL sterile EP tubes without an anticoagulant, and the serum obtained through centrifugation was stored at −20 °C. The heart, liver, spleen, lungs, and kidneys were rinsed with PBS and then weighed. Liver tissues for paraffin sections were fixed with 4% paraformaldehyde and stored at room temperature. Liver tissues for TEM were fixed with 2.5% glutaraldehyde and stored at 4 °C. Liver tissues for biochemical tests were stored at −20 °C. Liver tissues for qPCR were snap-frozen in liquid nitrogen and stored at −80 °C.

### 4.6. Organ Index

The final body weight before anesthesia and the weight of the organs harvested at the time of dissection were recorded, and the organ index was determined using the following formula [55]:

Organ index (%) = organ weight (g)/final body weight (g) × 100%

### 4.7. Histopathologic Observation of Liver

The liver tissue fixed with 4% paraformaldehyde was rinsed in running tap water for 1 h, dehydrated through an ethanol gradient, and cleared in xylene. The samples were then embedded in paraffin and cut into 4 μm thin slices. The sections were put into a constant-temperature-oven to be baked, deparaffinized in xylene, and rehydrated through a descending ethanol series. Hematoxylin and eosin (H&E) staining was performed according to standard protocols. After staining, the sections were dehydrated, cleared in xylene, and mounted. Histopathological changes were observed under a Leica DM1000 microscope (Leica, Heidelberg, Germany).

### 4.8. Ultrastructural Observation of Liver

Liver tissues prefixed with 2.5% glutaraldehyde were refixed with 1% osmium tetroxide, dehydrated step by step with different concentrations of acetone, and then embedded with Epon812. Then, ultrathin sections were made by positioning the semithin sections, stained successively with uranyl acetate and lead citrate. The ultrastructure was observed under a JEM-1400FLASH transmission electron microscope (JEOL, Tokyo, Japan).

### 4.9. Biochemical Test of Serum and Liver

The activities of ALT and AST in the serum were measured using a Hitachi 3100 automatic biochemical analyzer (HITACHI, Tokyo, Japan). The liver tissues used in biochemical tests were rinsed in pre-cooled PBS to make 10% liver tissue homogenates. The above biochemical kits were used to determine the activities of T-SOD, GSH-Px, and CAT and the MDA and total protein (TP) contents in the livers (Nanjing, China).

### 4.10. Serum ELISA Assay

The levels of ET, IFN-γ, TNF-α, IL-, IL-2, IgM, and IgG in the serum were detected by using the above ELISA kits (Shanghai, China).

### 4.11. Liver RNA Extraction and RT-qPCR Assay

The total liver RNA was extracted using the TRIzol method, and cDNA was synthesized using a PrimScript TM RT Reagent Kit and SYBR Premix Ex Taq TM II Kit (9108, Takara, Shiga, Japan). RT-qPCR was used to detect the TLR4, MyD88, NF-κB p65, IκBα, TNF-α, IL-1β, IL-6, NLRP3, and ASC expression levels, which were normalized by using β-actin as an internal reference gene, and the results were calculated using the 2^−ΔΔCt^ method. Primers were designed using PREMIER 5 (PREMIER Biosoft International, Palo Alto, CA, USA) and synthesized by Prime Biotech.

### 4.12. Data Analysis

All the data were analyzed using SPSS 27.0 (SPSS Inc., Chicago, IL, USA). A one-way ANOVA procedure was used to examine the statistically significant differences in the data, and multiple comparisons were performed using Duncan’s method. Graphs were plotted using GraphPad Prism 8.0.2 (GraphPad Software, San Diego, CA, USA) for graphing, and the results of the tests are presented as the means ± the standard deviations, with *p* < 0.05 indicating a significant difference and *p* < 0.01 indicating a highly significant difference.

## 5. Conclusions

This study explored the formulation design of a compound suspension containing paeoniflorin and curcumin, identifying the optimal formulation as follows: per 25 mL of suspension, 0.250 g paeoniflorin, 0.250 g curcumin, 0.250 g CMC-Na, 0.750 mL Tween 80, 0.300 g citric acid, and 0.025 g sodium benzoate. The suspension prepared with this formulation exhibited an orange-yellow hue, homogeneous turbidity, and excellent redispersibility. Significant protective effects against LPS/D-GalN-induced acute liver injury were observed, attributed to mechanisms involving enhanced antioxidant enzyme activities, inhibition of the TLR4/NF-κB/NLRP3 signaling pathway, and suppression of inflammasome assembly and release in hepatic tissues. Notably, this investigation focused solely on a fixed dosage (100 mg/kg BW for both compounds) and requires further systematic evaluation of different dose combinations to determine the optimal drug ratio.

## Figures and Tables

**Table 1:** Sedimentation volume ratio (F) and number of re-dispersions (N) for each group of suspensions.

Groups	1 h	2 h	3 h	6 h	1 Day	2 Days	3 Days	4 Days	5 Days	6 Days	7 Days	N
1	1.00	0.98	0.96	0.94	0.88	0.70	0.56	0.36	0.20	0.12	0.10	10
2	1.00	0.98	0.96	0.92	0.88	0.44	0.28	0.12	0.08	0.04	0.04	13
3	1.00	0.98	0.96	0.94	0.82	0.59	0.51	0.39	0.31	0.24	0.18	9
4	1.00	0.96	0.96	0.96	0.92	0.88	0.84	0.84	0.78	0.76	0.75	10
5	1.00	0.96	0.96	0.94	0.90	0.85	0.83	0.83	0.81	0.77	0.77	9
6	1.00	0.98	0.98	0.96	0.90	0.82	0.76	0.75	0.75	0.73	0.73	8
7	1.00	0.96	0.96	0.96	0.94	0.94	0.90	0.88	0.88	0.87	0.85	12
8	1.00	0.98	0.98	0.98	0.98	0.94	0.92	0.90	0.90	0.88	0.88	8
9	1.00	0.96	0.96	0.96	0.94	0.94	0.92	0.92	0.91	0.91	0.91	8

**Table 2:** Factors and levels in orthogonal test of suspension prescription.

Groups	CMC-Na/g	Tween 80/mL	Citric Acid/g	Sodium Benzoate/g
1	0.125	0.250	0.300	0.075
2	0.125	0.500	0.200	0.025
3	0.125	0.750	0.250	0.125
4	0.187	0.250	0.250	0.025
5	0.187	0.500	0.300	0.125
6	0.187	0.750	0.200	0.075
7	0.250	0.250	0.200	0.125
8	0.250	0.500	0.250	0.075
9	0.250	0.750	0.300	0.025

## Data Availability

The original contributions presented in this study are included in the article/Supplementary Materials. Further inquiries can be directed to the corresponding authors.

## Supplementary Materials

The following supporting information can be downloaded at https://www.mdpi.com/article/10.3390/ijms26136324/s1.
